# Phylogeny of *Acronychia* (Rutaceae) and First Insights into Its Historical Biogeography and the Evolution of Fruit Characters

**DOI:** 10.1371/journal.pone.0136296

**Published:** 2015-08-24

**Authors:** Laura Holzmeyer, Marco Duretto, Darren Crayn, Elvira Hörandl, Margaret Heslewood, Janani Jayanthan, Marc S. Appelhans

**Affiliations:** 1 Department of Systematics, Biodiversity and Evolution of Plants, University of Göttingen, Untere Karspüle 2, Göttingen, Germany; 2 National Herbarium of New South Wales, Royal Botanic Gardens & Domain Trust, Sydney, Australia; 3 Australian Tropical Herbarium, James Cook University, Cairns, Australia; 4 Department of Botany, Smithsonian Institution, Washington, DC, United States of America; University of Florida, UNITED STATES

## Abstract

**Background:**

The genus *Acronychia* (*Citrus* family, Rutaceae) contains 49 species of trees and shrubs that are found mainly in rain forest. The genus has a large distributional range from mainland southern Asia to Australia and New Caledonia, but most species are endemic to either New Guinea or Australia. This study aimed to provide the first detailed molecular phylogeny of *Acronychia* and use it to test the taxonomic value of fruit morphological characters, and infer the historical biogeography of the genus.

**Methodology:**

Phylogenetic analyses (Bayesian Inference, Maximum Likelihood) were undertaken on nucleotide sequence data from two plastid (*psbA-trnH*, *trnL-trnF*) and three nuclear markers (ETS, ITS, NIAi3) from 29 *Acronychia* species (59% of the genus) and representatives of related genera.

**Results and Conclusions:**

The results indicate that the South-East Asian genus *Maclurodendron* is nested phylogenetically within *Acronychia* and must be synonymized to render *Acronychia* monophyletic. Fruit morphological characters have been used previously to infer relationships within *Acronychia* and our analyses show that these characters are informative for some subclades but are homoplasious for the group as a whole. Apocarpous fruits are the ancestral state in *Acronychia* and subapocarpous and fully syncarpous fruits are derived. The unisexual flowers of *Maclurodendron* are derived from bisexual flowers, which are found in all species of *Acronychia* as well as its relatives. *Acronychia* probably first evolved on Australia with range expansion to New Guinea via stepping-stone dispersal or direct land connections within the Sahul Shelf, followed by two independent dispersals to areas west of New Guinea. Most species of *Acronychia* occur in either Australia or New Guinea, but no species occurs in both regions. This is surprising given the close proximity of the landmasses, but might be explained by ecological factors.

## Introduction

*Acronychia* belongs to a clade of mainly Australasian rain forest genera in the citrus family, Rutaceae [1, 2]. The 49 accepted species in the genus may be shrubs or trees up to 30 m tall [3, 4, 5] with leaves simple or trifoliolate (both character states may occur within a single species), flowers tetramerous with eight stamens, and the gynoecium developing into a syncarpous (sometimes only at base) and drupaceous fruit. While the overall habit and the flowers are similar to its close relatives *Euodia*, *Medicosma*, *Melicope* and *Tetractomia*, these genera differ from *Acronychia* in having capsular or follicular fruits [3, 4, 6, 7, 8].

The centers of species-richness and endemism of *Acronychia* are New Guinea and eastern Australia, with 27 endemic species in the former and 19 endemic species in the latter [3, 4, 5, 9]. Very few species occur outside of this area and the relatively large distribution area of the genus as a whole is mainly due to two species: *A*. *pedunculata*, which is distributed in southern Asia from India to Taiwan and throughout the Malesian region; and *A*. *trifoliolata*, which occurs from Java and Sulawesi to the Solomon Islands. One species, *A*. *laevis*, is widespread in eastern Australia and also occurs in New Caledonia; although recorded from Lord Howe Island (e.g., [3]) the species is now not considered to occur there [4, 10]. The altitudinal range of the genus is from sea level to about 3000 m [3, 4].

*Acronychia* has been subdivided into several ‘evolutionary lines’ (without taxonomic rank), based on the presence or absence of septicidal fissures in the gynoecium and the structure of the exocarp and mesocarp [3]. Fruits with septicidal fissures and a (sub)fleshy exo- and mesocarp have been regarded as ‘primitive’ in *Acronychia*, and fully syncarpous fruits with a (sub)woody mesocarp and fleshy exocarp as derived [3]. This hypothesis has not yet been tested using cladistic methods. *Acronychia octandra* is unique in the genus in having almost fully apocarpous and beaked carpels and it has been regarded as ‘…an early stage in the evolution of the genus.’ ([11] p.445). *Acronychia octandra* was initially described as *Euodia octandra* and only transferred to *Acronychia* recently by Hartley [11]. The combination of beaked and apocarpous drupes as well as diplostemonous flowers (sometimes with four stamens reduced to staminodes) is otherwise found in the mainly New Caledonian genera *Comptonella*, *Picrella* and *Sarcomelicope* [12, 13, 14] making the placement of *A*. *octandra* within *Acronychia* doubtful.

Due to similarities in vegetative and floral morphology the genera *Acronychia*, *Euodia* and *Melicope* have a long history of confusion and many species of these genera have a synonym in one or both of the other genera. In fruit morphology, however, these three genera are strikingly different which led Engler [15] to separate them at subfamily level: *Acronychia* was placed in subfamily Toddalioideae, which was characterized by indehiscent fruits, and *Euodia* and *Melicope* in the dehiscent-fruited Rutoideae. Most botanists in the 20^th^ century largely adopted Engler´s system (e.g., [16, 17, 18]), until analyses of secondary compounds provided evidence for the artificiality of the system [19, 20, 21, 22]. Molecular phylogenetic studies supported the chemosystematic studies and revealed that Rutoideae and Toddalioideae are largely intermixed, and that Aurantioideae is the only non-monogeneric subfamily that can be regarded as monophyletic [23, 24, 25, 26, 27, 28, 29, 30].

The close relationship between *Acronychia* and its dehiscent-fruited relatives *Euodia* and *Melicope* is a prime example of the artificiality of Engler´s classification of Rutaceae [1, 26]. In addition to *Euodia* and *Melicope*, a group of about 30 genera mainly from Asia and Australia, the so-called *Euodia*-alliance, are possible close relatives of *Acronychia* [31]. Most of these genera have been sampled in recent molecular analyses [1, 2], which showed that the *Euodia*-alliance is not monophyletic.

The genus *Acronychia* itself is probably not monophyletic–*Maclurodendron* is nested within it [1]. *Maclurodendron* was erected by Hartley [32] for three South-East Asian species he earlier ([3] p. 469) excluded from *Acronychia*. The genus now consists of six species, which are distributed on the South-East Asian mainland, and from Sumatra and the Malay Peninsula east to the Philippines [32]. *Maclurodendron* is morphologically very similar to *Acronychia* and the only consistent differences between them are dioecious (*Maclurodendron*) versus hermaphroditic (*Acronychia*) flowers and imbricate (*Maclurodendron*) versus valvate (*Acronychia*) petals [32].

In this study we present the first detailed phylogenetic reconstruction of the genus *Acronychia* based on nuclear and plastid sequences from 59% of the species selected to cover the geographical range and morphological diversity of the genus. The major goals of this study were to (1) reconstruct the phylogenetic history of *Acronychia*, (2) identify the closest relatives of *Maclurodendron* within *Acronychia* in order to gain insight into the evolution of dioecy in the group and the geographic origin of *Maclurodendron*, (3) determine whether or not *A*. *octandra* is part of *Acronychia* despite its unusual fruit morphology, (4) evaluate the relevance of Hartley´s [3] evolutionary lines that are mainly based on the presence or absence of septicidal fissures, and the structure of the exo- and mesocarp for classification within *Acronychia*, and to (5) identify biogeographic patterns, and infer directions of dispersal and the geographical origin of the genus.

## Materials and Methods

### Taxon Sampling

In total, 29 of the 49 currently accepted species of *Acronychia*, which together comprehensively sample the morphological diversity and geographical range of the genus, were included in this study. These 29 species include 15 of the 19 Australian endemics (79%) and 10 of the 27 New Guinean endemics (37%). The sparser sampling of the latter is due to most species (52%) being known only from the type collection or from very few collections from the type locality, which is, in many cases, quite remote [3, 5, 9]. All widespread species (viz. extending beyond Australia and New Guinea) were sampled. *Acronychia laevis*, which occurs in eastern Australia and New Caledonia, was sampled from Australian material. One sample of the genus *Maclurodendron*, which was shown to belong to *Acronychia* [1], was included in the analysis. *Euodia* has been shown to be part of the clade sister to the main *Melicope*/*Acronychia* clade of Appelhans et al. [1] and so two of the seven species of *Euodia* (*E*. *hortensis* and *E*. *montana*) were used as outgroups. Several species of *Melicope*, *Medicosma*, *Tetractomia* and *Comptonella*, the closest relatives of *Acronychia* [1], were included to assess the monophyly of the latter.

Voucher information for all specimens used in this study is given in Tables 1 and 2.

**Table 1:** Voucher information and Genbank accession numbers for specimens used in the combined analyses. Herbarium acronyms are according to Index Herbariorum (http://sweetgum.nybg.org/ih/). *A* = *Acronychia*; *C* = *Comptonella*; *E* = *Euodia*; *M* = *Melicope*; *Ma* = *Maclurodendron*; *Me* = *Medicosma*; *T* = *Tetractomia*. An asterisk (*) indicates sequences that were obtained in this study.

	Voucher information	
Taxon	Collector & number (Herbarium)	Origin	*trnL-trnF*	ITS	ETS	*psbA-trnH*	NIAi3
*A*. *acronychioides*	*Forster PIF30987* (L)	Australia, Queensland	LN849177*	LN849136*	LN849220*	-	-
*A*. *acuminata*	*Ford 3997* (CNS)	Australia, Queensland	LN849178*	LN849137*	LN849221*	LN849160*	LN849199*
*A*. *baeuerlenii*	*Beesley 1080a* (NSW)	Australia, NSW	LN849179*	LN849138*	LN849222*	LN849161*	LN849200*
*A*. *baeuerlenii*	*Rossetto ABNIG1* (NSW)	Australia, NSW	LN849180*	LN849139*	LN849223*	LN849162*	LN849201*
*A*. *brassii*	*Appelhans 454* (LAE, US)	Papua New Guinea	HG971153	HG971304	HG971458	HG971025	HG971612
*A*. *brassii*	*Appelhans 466* (LAE, US)	Papua New Guinea	HG971154	HG971305	HG971459	HG971026	HG971613
*A*. *brassii*	*Appelhans 467* (LAE, US)	Papua New Guinea	HG971155	HG971306	HG971460	HG971027	HG971614
*A*. *chooreechillum*	*Telford 11393* (NSW)	Australia, Queensland	LN849181*	LN849141*	LN849226*	LN849163*	LN849202*
*A*. *eungellensis*	*Forster PIF25513* (CNS)	Australia, Queensland	-	-	LN849228*	LN849164*	LN849203*
*A*. *imperforata*	*Forster PIF30952* (L)	Australia, Queensland	LN849182*	LN849143*	LN849231*	-	LN849204*
*A*. *laevis*	*Forster PIF30953* (L)	Australia, Queensland	LN849183*	LN849144*	LN849232*	-	-
*A*. *ledermannii*	*Appelhans 426* (LAE, US)	Papua New Guinea	HG971156	HG971307	HG971461	HG971028	HG971615
*A*. *ledermannii*	*Appelhans 448* (LAE, US)	Papua New Guinea	HG971157	HG971308	HG971462	HG971029	HG971616
*A*. *ledermannii*	*Appelhans 458* (LAE, US)	Papua New Guinea	HG971158	HG971309	HG971463	HG971030	HG971617
*A*. *littoralis*	*Rossetto ALBAL1* (NSW)	Australia, NSW	-	AY588597	LN849233*	LN849165*	LN849205*
*A*. *littoralis*	*Rossetto ALAB1* (NSW)	Australia, NSW	LN849184*	-	LN849234*	LN849166*	LN849207*
*A*. *littoralis*	*Rossetto ALSC2* (NSW)	Australia, NSW	LN849185*	-	LN849235*	LN849167*	LN849206*
*A*. *murina*	*Utteridge 542* (A)	Papua New Guinea	LN849186*	LN849145*	LN849236*	-	-
*A*. *murina*	*Regalado 1023* (A)	Papua New Guinea	LN849187*	LN849146*	LN849237*	LN849168*	LN849209*
*A*. *murina*	*Takeuchi 24793* (A)	Papua New Guinea	LN849188*	LN849147*	LN849238*	-	LN849208*
*A*. *oblongifolia*	*Rossetto AOB1* (NSW)	Australia, NSW	LN849189*	LN849148*	LN849239*	LN849169*	LN849210*
*A*. *oblongifolia*	*Winsbury 97* (CBG)	Australia, NSW	EU493242	EU493185	HG971464	EU493204	-
*A*. *octandra*	*Forster PIF34176* (MEL)	Australia, Queensland	LN849190*	LN849149*	LN849240*	LN849170*	-
*A*. *parviflora*	*Ford 4434* (CNS)	Australia, Queensland	LN849191*	LN849150*	LN849241*	-	LN849211*
*A*. *pauciflora*	*Rossetto APAWIL1* (NSW)	Australia, NSW	LN849192*	LN849151*	LN849242*	LN849171*	LN849212*
*A*. *pedunculata*	*Brambach 1503* (GOET)	Sulawesi	LN849193*	LN849152*	LN849243*	-	LN849214*
*A*. *pedunculata*	*Wen 12364* (US)	Java	-	LN849153*	LN849244*	LN849172*	LN849213*
*A*. *pedunculata*	*de Wilde 6834* (L)	Thailand	HG002754	HG002398	HG002527	HG002652	HG002957
*A*. *pubescens*	*Rossetto APUWIL1* (NSW)	Australia, NSW	LN849194*	LN849154*	-	LN849173*	LN849215*
*A*. *pullei*	*Appelhans 460* (US)	Papua New Guinea	HG971159	HG971310	HG971465	HG971031	HG971618
*A*. *reticulata*	*Coode 8081* (L)	Indonesia, Papua	HG971160	HG971311	HG971466	-	-
*A*. *suberosa*	*Forster PIF28797* (L)	Australia, Queensland	LN849195*	LN849155*	LN849246*	-	-
*A*. *suberosa*	*Rossetto ASNIG1* (NSW)	Australia, NSW	LN849196*	LN849156*	LN849247*	LN849174*	LN849216*
*A*. *trifoliolata var*. *microcarpa*	*James 459* (LAE, BISH, GOET)	Papua New Guinea	HG971161	HG971312	HG971467	HG971032	HG971619
*A*. *trifoliolata var*. *microcarpa*	*Appelhans 416* (LAE, US)	Papua New Guinea	HG971162	HG971313	HG971468	HG971033	HG971620
*A*. *vestita*	*Forster PIF27548* (L)	Australia, Queensland	-	LN849157*	LN849248*	-	LN849217*
*A*. *wilcoxiana*	*Rossetto AWIL1* (NSW)	Australia, NSW	LN849197*	LN849158*	LN849249*	LN849175*	LN849218*
*C*. *microcarpa*	*Lowry 5734* (MO)	New Caledonia	HG971275 + HG971287	HG971319	HG971473	HG971036	HG971624
*E*. *hortensis*	*Drake 235* (US)	Polynesia, Tonga	HG002786 + HG002862	HG002399	HG002528	HG002653	HG002958
*E*. *montana*	*James 381* (LAE, BISH, GOET)	Papua New Guinea	HG971170	HG971327	HG971480	HG971043	HG971630
*M*. *clusiifolia*	*Wagner 6912* (US)	Hawaii	HG002796 + HG002872	HG002410	HG002540	HG002661	HG002969
*M*. *durifolia*	*Appelhans 424* (US)	Papua New Guinea	HG971195	HG971360	HG971512	HG971068	HG971657
*M*. *elleryana*	*Appelhans 413* (LAE, US)	Papua New Guinea	HG971207	HG971372	HG971524	HG971080	HG971669
*M*. *ternata*	*Appelhans 487* (GOET)	Cultivated Botanical Garden Göttingen	HG971258	HG971432	HG971585	HG971130	HG971722
*Ma*. *porteri*	*John 145743* (L)	Malaysia, Sabah	HG971289	HG971329	HG971483	-	-
*Me*. *glandulosa*	*Forster 25045* (L)	Australia, Queensland	HG971172	HG971330	HG971484	HG971045	-
*T*. *tetrandrum*	*Utteridge 436* (L)	Indonesia, Papua	LN849198*	LN849159*	LN849250*	LN849176*	LN849219*
*T*. *tetrandrum*	*Beaman 8917* (L)	Borneo	HG971271	HG971449	HG971602	HG971145	HG971732

**Table 2:** Voucher information and Genbank accession numbers for specimens additionally used for the separate ETS and ITS analyses. Herbarium acronyms are according to Index Herbariorum (http://sweetgum.nybg.org/ih/). *A* = *Acronychia*. An asterisk (*) indicates sequences that were obtained in this study.

	Voucher Information			
Taxon	Collector & number (Herbarium)	Origin	ITS	ETS
*A*. *acronychioides*	*Elick 283* (CNS)	Australia, Queensland	DQ225819	*-*
*A*. *cartilaginea*	*Takeuchi 23857* (A)	Papua New Guinea	LN849140*	LN849224*
*A*. *chooreechillum*	*Hyland 13750* (L)	Australia, Queensland	*-*	LN849225*
*A*. *crassipetala*	*Elick 279* (CNS)	Australia, Queensland	DQ225818	*-*
*A*. *emarginata*	*Takeuchi 20017* (A)	Papua New Guinea	*-*	LN849227*
*A*. *foveata*	*Takeuchi 23784* (A)	Papua New Guinea	*-*	LN849229*
*A*. *foveata*	*Takeuchi 23863* (A)	Papua New Guinea	LN849142*	LN849230*
*A*. *laevis*	*Elick 278* (CNS)	Australia, Queensland	DQ225817	*-*
*A*. *pedunculata*	*Poon & Woo R5* (CUHK)	*No info in Genbank*	DQ225816	*-*
*A*. *rugosa*	*Milliken 1405* (A)	Indonesia, Papua	-	LN849244*
*A*. *vestita*	*Elick 286* (CNS)	Australia, Queensland	DQ225820	*-*

### DNA extraction, amplification and sequencing

Laboratory work was carried out in Cairns, Göttingen and Sydney. Leaf samples were taken from silica-dried specimens or from herbarium sheets. The plant material was ground in a TissueLyser II (QIAGEN, Valencia, California, USA) using stainless steel beads. Total DNA was extracted using the Qiagen DNeasy Plant Mini Kit (QIAGEN, Valencia, California, USA) according to the manufacturer´s instructions (Cairns, Sydney). In the Göttingen laboratory, the original protocol was modified in two ways: the extraction was done without RNase and the lysis step was increased from 10 to 30 minutes to increase the efficiency of cell wall lysis.

Five markers were amplified and sequenced for this study as these markers proved to be highly variable and informative in the closely related genus *Melicope* [1, 33]. The three markers chosen from the nuclear genome were ITS (internal transcribed spacer), ETS (external transcribed spacer) and NIAi3 (the third intron from the single copy gene NIA; nitrate reductase) and the spacer regions *trnL-trnF* and *psbA-trnH* were chosen from the plastome.

PCR was performed in a 25μL reaction volume containing 2.5 μL of 10x PCR buffer, 2.5 mM MgCl_2_, 0.2 mM of each dNTP, 0.2 μM of each primer, 3 U of *BioTaq* (Bioline, London, UK) and 1 μL of template DNA. The amplification program used for ETS, ITS, *psbA-trnH* and *trnL-trnF* consisted of 5 minutes of initial denaturation at 95°C; 36 cycles of 1 minute denaturation at 95°C, 40 seconds of annealing at 53–54°C, an elongation of 40 seconds to 1 minute (depending on the length of the target sequence) at 72°C; and a final elongation for 5 minutes. A touchdown PCR program was used for the amplification of NIAi3 [34].

The following primers were used for PCR and for Sanger sequencing: Bur1 & 18S-IGS [35, 36] or myrtF and ets18s [37, 38] for ETS; ITS2, ITS3, ITS4, ITS5 & ITS5A [39, 40] for ITS; NIAi3_RutaF & NIAi3_RutaR [33] for NIAi3; psbA, and trnH [41] or trnH2 [42] for *psbA-trnH*; and C, D, E & F for *trnL-trnF* [43].

Amplified PCR products were cleaned using either the MSB Spin PCRapace Kit (Göttingen laboratory; Stratec Biomedical AG, Birkenfeld, Germany) or the SureClean Plus Kit (Cairns and Sydney laboratories; BioLine, London, UK) and then sequenced using the BigDye version 3.1 kit (Applied Biosystems, Foster City, California, USA) following the manufacturer´s instructions. The sequencing products were purified using ethanol precipitation and run on ABI 3100 (Göttingen) or ABI 3730 (Cairns, Sydney) sequencers.

Genbank accession numbers for all sequences used in this study are shown in Tables 1 and 2.

### Multiple sequence alignment and phylogenetic reconstruction

Sequences were checked and edited in Geneious version 5.6.4 (Biomatters Ltd, Auckland, New Zealand) and then aligned in SATé [44] using the settings described in Appelhans et al. [33]. The alignments were checked and manually edited in Geneious version 5.6.4 and MacClade 4.08 (Sinauer Associates Inc., Sunderland, MA, USA), and are available from TreeBASE (study accession URL: http://purl.org/phylo/treebase/phylows/study/TB2:S17897). jModeltest v2.1.3 was used to determine the best-fitting model of sequence evolution for each single marker alignment under the Bayesian Information Criterion (BIC; [45, 46]). The best-fitting models are shown in Table 3.

**Table 3:** Models of sequence evolution estimated using the Bayesian Information Criterion (BIC) algorithm in jModeltest 2.1.3. The table shows the models with the highest likelihood scores and the highest available models that are available in the programs MrBayes and Garli.

	*trnL-trnF*	ITS	ETS	*psbA-trnH*	NIAi3
Best BIC model	TPM1uf+G	TrNef+G	TPM3uf+G	F81+G	K80+G
Best BIC model available for MrBayes	HKY+G	SYM+I	HKY+G	F81+G	K80+G
Best model available for Garli	TVM+G	TrNef +G	TrN+G	F81+G	K80+G

Phylogenetic tree estimations were performed using Bayesian Inference (MrBayes 3.2.1; [47, 48]) and Maximum Likelihood (ML; Garli 2.0; [49]). All single marker alignments were first analyzed separately and, since the trees showed no supported incongruences, the alignments of the five markers were concatenated and analyzed together. In order to evaluate the influence of the hybrid species *A*. *littoralis* [50] on the tree topology, we performed an additional Bayesian analysis excluding the *A*. *littoralis* specimens. Only specimens for which sequences of at least three out of the five markers could be obtained were included in these concatenated analyses in order to minimize the amount of missing data. Overall, sequencing of ETS and ITS had a higher success-rate and several species that were not included in the concatenated analyses were included in the ETS and/or ITS analyses. We therefore show and discuss the results of the ETS and ITS analyses in addition to those of the concatenated analyses.

The Bayesian analysis of the single marker datasets and the concatenated dataset consisted of two independent Markov Chain Monte Carlo (MCMC) runs with four chains each. The length of the runs was set to ten million generations and a tree was saved every 100th generation. In order to evaluate if the two runs reached stationarity, the average standard deviation of split frequencies between the two runs was checked; a value of <0.01 was regarded as sufficient. The effective sample size (ESS) and the burn-in were determined using Tracer v1.6.0 [51]. Based on the results the first 10–15% of the trees were discarded as burn-in and the remaining trees were used to calculate a 50% majority-rule consensus tree in MrBayes 3.2.1.

The ML analyses consisted of five independent searches each comprising 1000 bootstrap replicates to obtain statistical support (bootstrap values, BS) for the tree topology. The models of sequence evolution used for the ML analyses were estimated as described above and are shown in Table 3. SumTrees v3.3.1, as implemented in the python package DendroPy 3.12.0 [52], was used to construct the bootstrap consensus tree.

The consensus trees from the Bayesian and ML analyses were edited in FigTree v1.4.0 (http://tree.bio.ed.ac.uk/software/figtree/) and rooted using the two *Euodia* species as outgroups. Clades with Bayesian posterior probability (PP) values of ≥0.95 were regarded as statistically supported. In the ML analyses, clades with a bootstrap (BS) value of 90% or higher were regarded as strongly supported, clades with a BS support of 70%–89% were regarded as having medium support, and clades with a BS support of 50%–69% were considered to have low support. Clades with less than 0.95 PP and 50% BS were regarded as not supported and treated as polytomies.

### Tracing morphological characters and geographical areas

Two fruit characters—fusion of carpels, and texture of mesocarp—have previously been used to define ‘evolutionary lines’ within *Acronychia* [3]. The reliability of these characters as phylogenetic markers was critically assessed by plotting them on the Bayesian consensus tree of the concatenated matrix using parsimony in Mesquite v.3.02 [53]. Fruit character states were coded as follows: a) degree of fusion of carpels: 0- fruit completely syncarpous; 1- septicidal fissures extending from ¼ to ½ the length of the fruit; 2- septicidal fissures extending to at least ½ of the length of the fruit; 3- fruit nearly completely apocarpous (and carpels beaked); 4- other fruit types (dehiscent fruits in most taxa and drupaceous fruits with varying degrees of carpel fusion in *Comptonella*); b) texture of the mesocarp: 0- mesocarp drying semifleshy and/or not differing from exocarp; 1- mesocarp drying spongy-crustaceous; 2- mesocarp drying (sub)woody; 3- no evident mesocarp; 4- other fruit types. Most outgroup taxa have dry and dehiscent fruits, so that the texture of the mesocarp is not comparable to that of drupaceous fruits, and the fruits of *Comptonella* and *Melicope* can be fully syncarpous or almost apocarpous. In these cases the characters state ‘other fruit types’ was used.

To reconstruct ancestral areas and the geographical origin of *Acronychia*, geographical areas were plotted on the Bayesian consensus tree of the concatenated matrix using Mesquite with the settings described above. More sophisticated ancestral area reconstruction methods implemented in programs such as Lagrange [54, 55] or BioGeoBEARS [56] require fully bifurcating and dated phylogenies. Since no fossils have been described for *Acronychia* or near relatives, such analyses are only feasible for a broader taxon sampling (Appelhans et al., in preparation) and are thus beyond the scope of this study. Geographic areas were coded as follows: 0- Eastern Australia; 1- New Guinea; 2- widespread. Several outgroup taxa are narrow endemics, e.g. *Comptonella microcarpa* (New Caledonia) and *Melicope clusiifolia* (Hawaiian Islands). However, both taxa were coded as having a widespread distribution since they are the sole representatives in this analysis of clades with wide distributions in the South-East Asian and Pacific areas [1].

## Results

### Phylogeny and Systematics

**Fig 1:**
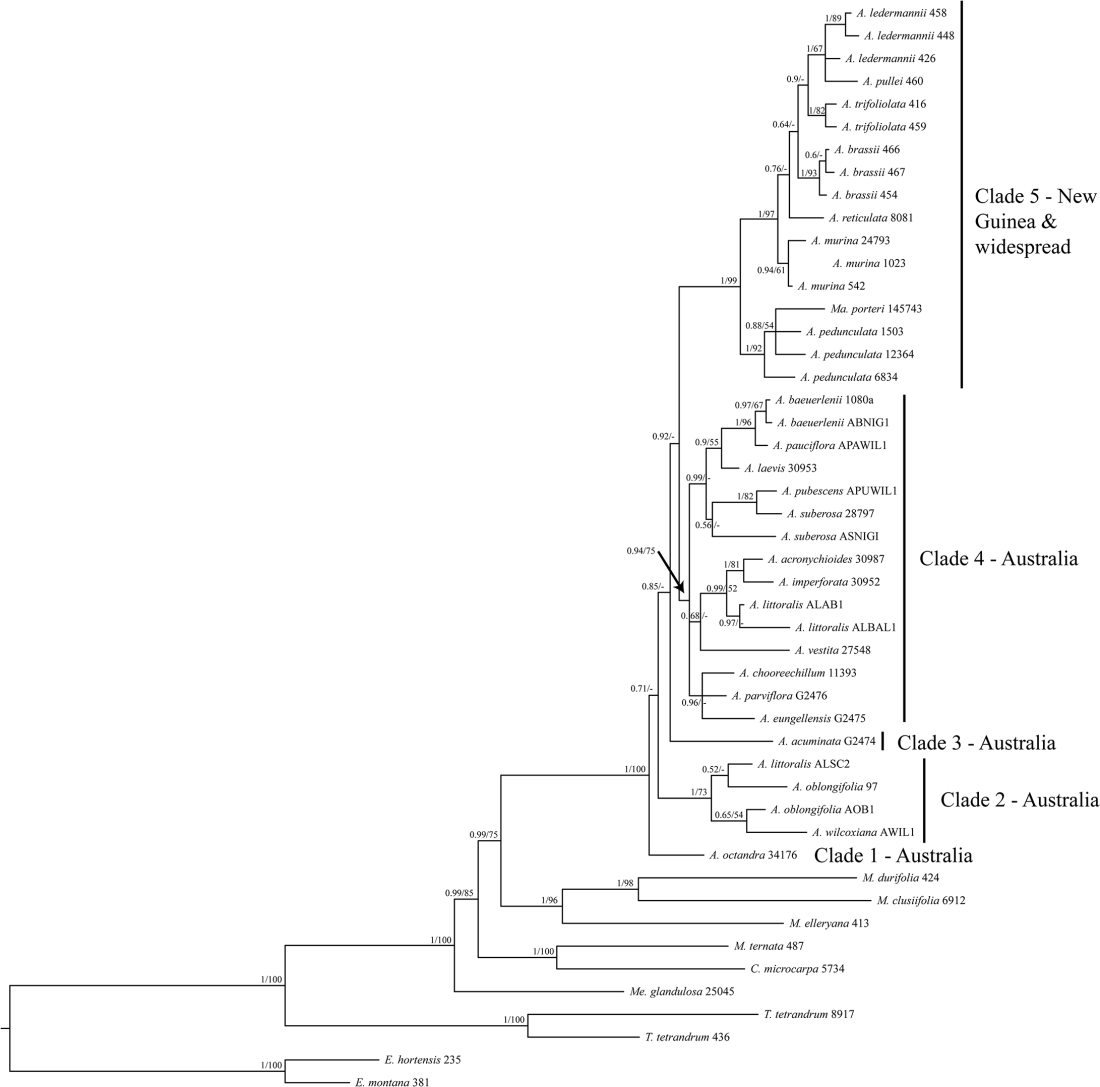
Phylogram of the 50% majority-rule consensus tree of the Bayesian analysis of the concatenated data set of ETS, ITS, NIAi3, *psbA-trnH* and *trnL-trnF* sequences. Posterior probability (PP) values of the Bayesian analysis and bootstrap values (BS) of the Garli analysis are displayed above the branches and unsupported nodes are marked with a hyphen (-). The voucher number is displayed after the species name for all taxa. *A* = *Acronychia*; *C* = *Comptonella*; *E* = *Euodia*; *M* = *Melicope*; *Ma* = *Maclurodendron*; *Me* = *Medicosma*; *T* = *Tetractomia*.

**Fig 2:**
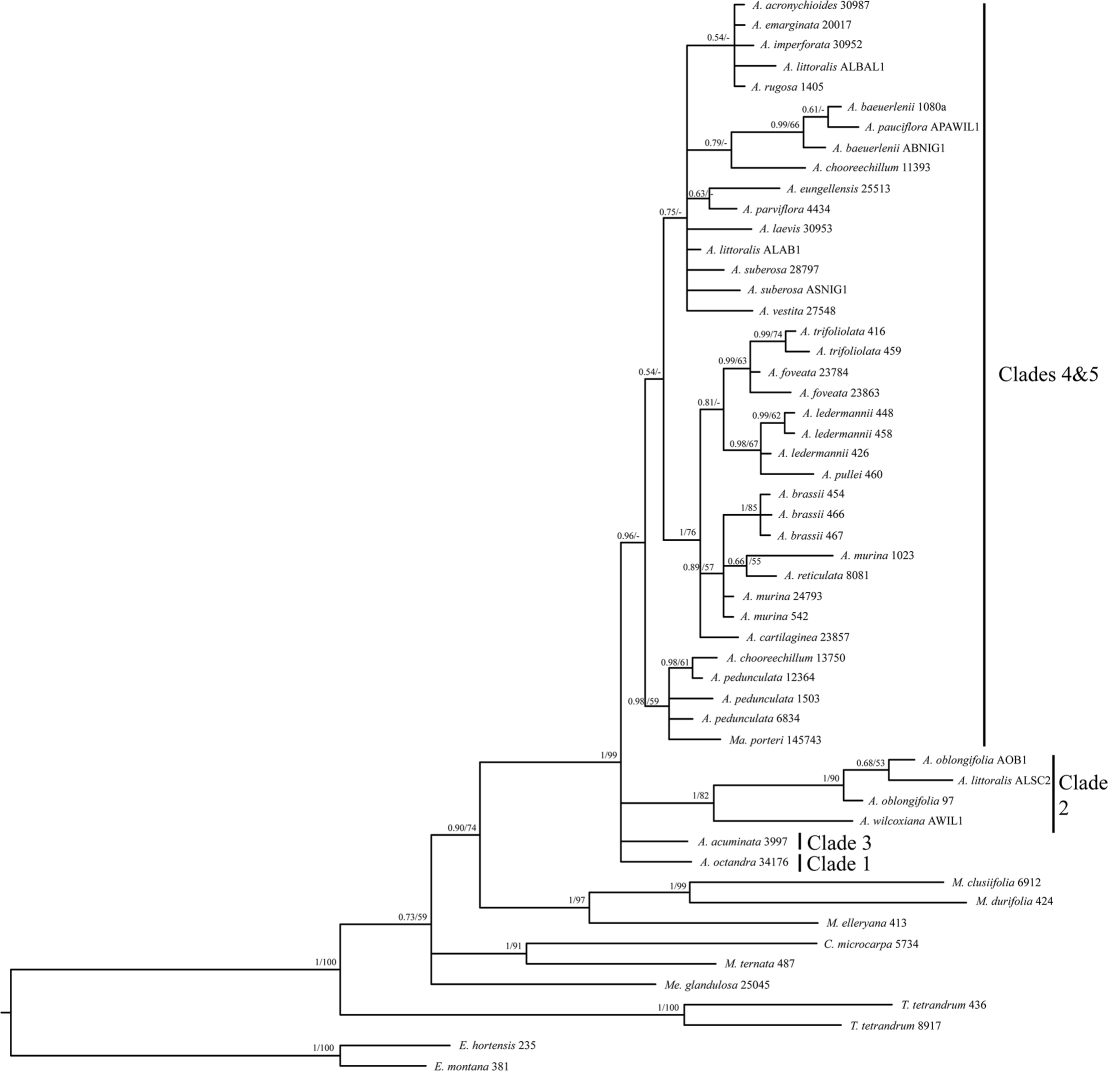
Phylogram of the 50% majority-rule consensus tree of the Bayesian analysis based on the ETS dataset. Posterior probability (PP) values of the Bayesian analysis and bootstrap values (BS) of the Garli analysis are displayed above the branches and unsupported nodes are marked with a hyphen (-). The voucher number is displayed after the species name for all taxa. The clade numbers refer to the clades from Fig 1. *A* = *Acronychia*; *C* = *Comptonella*; *E* = *Euodia*; *M* = *Melicope*; *Ma* = *Maclurodendron*; *Me* = *Medicosma*; *T* = *Tetractomia*.

**Fig 3:**
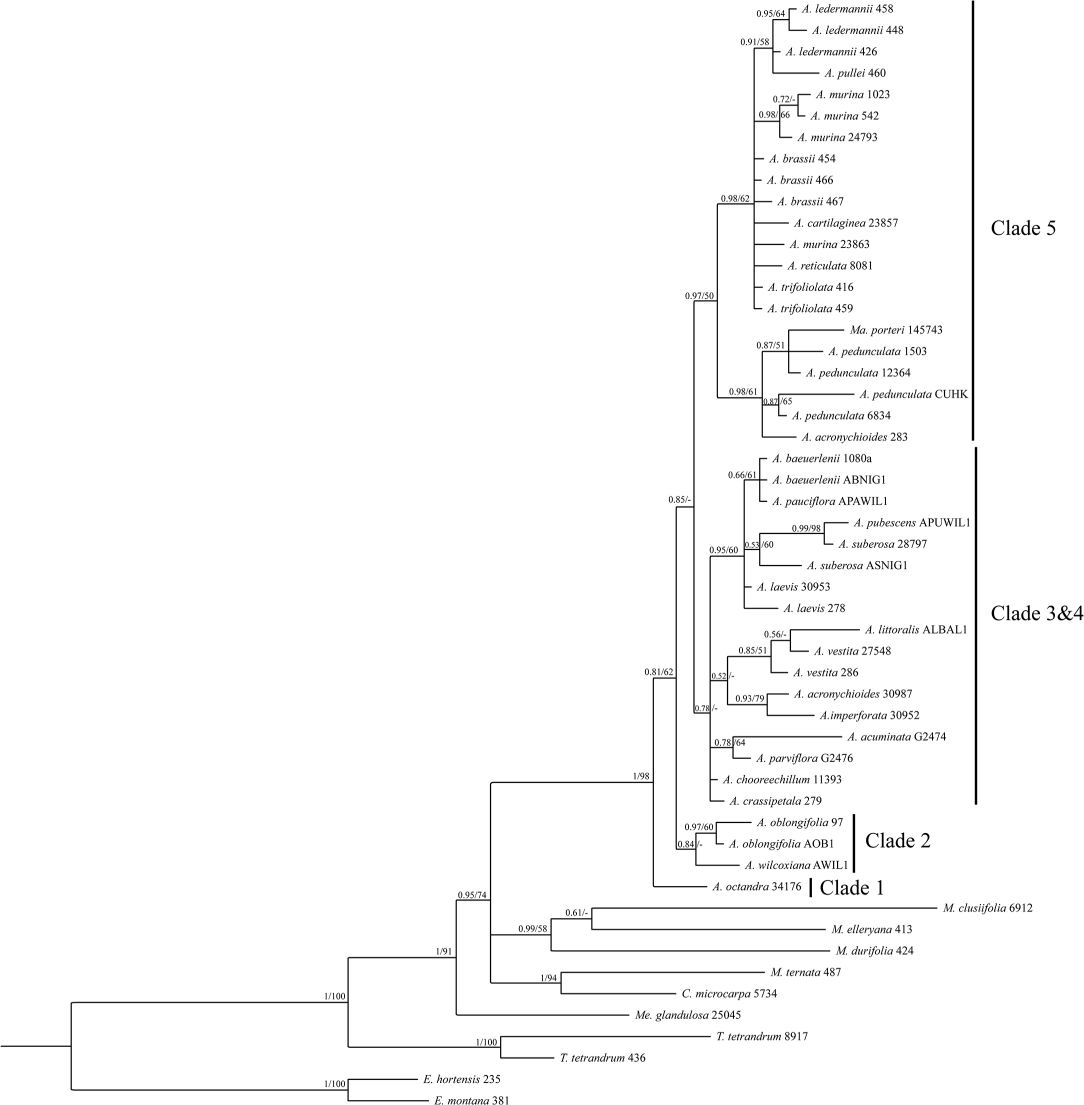
50% majority-rule consensus tree of the Bayesian analysis of the ITS dataset. Posterior probability (PP) values of the Bayesian analysis and bootstrap values (BS) of the Garli analysis are displayed above the branches and unsupported nodes are marked with a hyphen (-). The voucher number is displayed after the species name for all taxa. The clade numbers refer to the clades from Fig 1. *A* = *Acronychia*; *C* = *Comptonella*; *E* = *Euodia*; *M* = *Melicope*; *Ma* = *Maclurodendron*; *Me* = *Medicosma*; *T* = *Tetractomia*.

The consensus trees from the Bayesian and the ML analyses of the concatenated five-marker dataset (Fig 1) are generally well resolved, well supported, and showed no supported incongruences. The backbone of the trees is highly supported with most clades showing high support of ≥0.95 PP and ≥90% BS respectively. Consensus trees from the single-marker analyses are generally less well resolved (ETS, Fig 2; ITS, Fig 3; trees for other markers not shown), but showed no supported incongruences (incongruent clades with ≥0.95 PP and ≥70% BS) with the consensus trees of the concatenated dataset.

Our results confirm that the genus *Maclurodendron* is nested within *Acronychia*, indicating that *Maclurodendron* needs to be synonymized under *Acronychia* for *Acronychia* to be monophyletic. The *Acronychia* clade (incl. *Maclurodendron*) is sister to a clade composed of *Melicope clusiifolia*, *M*. *durifolia*, and *M*. *elleryana*, which represent the two largest sections of *Melicope*: *Lepta* and *Pelea*. The *Acronychia*-*Pelea*-*Lepta* clade in turn is sister to a clade consisting of *M*. *ternata*, the type of *Melicope*, and the New Caledonian endemic genus *Comptonella*.

All these main clades are highly supported (0.99–1.00 PP and 90–100% BS) which supports the finding of Appelhans et al. [1] that *Melicope* is paraphyletic with respect to *Acronychia*, *Comptonella* and *Maclurodendron*.

Within *Acronychia*, five distinct clades are observed. Clade 1, consisting solely of the Australian species *A*. *octandra*, is sister to the remainder of *Acronychia* (0.71 PP, not supported in the ML analysis). Clade 2 (1.00 PP, 73% BS) contains samples of *A*. *littoralis*, *A*. *oblongifolia* and *A*. *wilcoxiana*, species which are also exclusively Australian. Clade 3 comprises *A*. *acuminata*, a rare, narrowly endemic species of North-East Queensland (Australia). Its position as sister to Clades 4 and 5 is resolved only by the Bayesian analysis, but without support (0.85 PP). Clade 4 is entirely Australian except for *A*. *laevis*, which is also present on New Caledonia. Clade 4 is resolved by both Bayesian and ML analyses, but is supported only in the ML analysis (0.94 PP, 75% BS). This large clade is further divided into three subclades. Clade 5 (1.00 PP, 99% BS) lacks Australian taxa and consists of all sampled New Guinean species, the two widespread species *A*. *pedunculata* and *A*. *trifoliolata*, and the genus *Maclurodendron*. Within this clade, *A*. *pedunculata* and *Maclurodendron* form a well-supported subclade (1.00 PP, 92% BS) that is sister to the rest of the clade. The second widely distributed species, *A*. *trifoliolata*, is most closely related to *A*. *ledermannii* and *A*. *pullei* (0.90 PP, not supported in the ML analysis), two rather widely distributed New Guinean endemics.

For several species more than one sample was included. Three of these species were found to be non-monophyletic with high support in both Bayesian and ML analyses. The three specimens of *A*. *ledermannii* form a clade with *A*. *pullei*. *Acronychia suberosa* was resolved as paraphyletic with *A*. *pubescens*. *Acronychia littoralis* was shown to be polyphyletic with two accessions in Clade 4 (related to *A*. *vestita*, *A*. *imperforata* and *A*. *acronychioides*) and one accession in Clade 2 (related to *A*. *oblongifolia* and *A*. *wilcoxiana*). In addition, *A*. *pedunculata* was resolved as paraphyletic with respect to *Maclurodendron*, although this topology was not supported in the Bayesian analysis (0.88 PP) and only weakly supported in the ML analysis (54% BS).

The additional Bayesian analysis of the concatenated data matrix excluding the samples of the hybrid species *A*. *littoralis* revealed one difference in the tree topology. The two accessions of *A*. *oblongifolia* were resolved as a clade with 0.95 PP, which was sister to *A*. *wilcoxiana* with 1.00 PP (results not shown), while one of the accessions of *A*. *oblongifolia* was sister to *A*. *wilcoxiana* in the analysis including *A*. *littoralis*. The support values remained largely unchanged.

For several species only ETS and/or ITS sequences were obtained and they were not included in the combined analyses in order to minimize the effect of missing data on the results. The assignment of these species to clades within *Acronychia* was done using separate ETS and ITS analyses (Figs 2 and 3). In the ETS analysis (Fig 2) the two New Guinean species–*A*. *foveata* and *A*. *cartilaginea*–group with New Guinean species (Clade 5 in Fig 1). *Acronychia foveata* is most closely related to the widespread *A*. *trifoliolata*. Two other New Guinean species–*A*. *emarginata* and *A*. *rugosa*–group with the large Australian clade (Clade 4 in Fig 1), however, this placement was only supported in the ML consensus tree of ETS. Two Genbank accessions from Australia representing the species *A*. *acronychioides* and *A*. *crassipetala* were included in the ITS dataset. While *A*. *crassipetala* is part of Clade 4, the Genbank accession of *A*. *acronychioides* clusters with *A*. *pedunculata*, which is the most widespread species of *Acronychia*, but does not occur in Australia. The identity of the voucher (Elick 283, CNS) for the *A*. *acronychioides* ITS sequence has been confirmed by two of us (DC, MSA), but this surprising placement should be tested with data obtained in a different laboratory from additional, independent samples.

### Fruit Evolution and Hartley´s Evolutionary Lines

**Fig 4:**
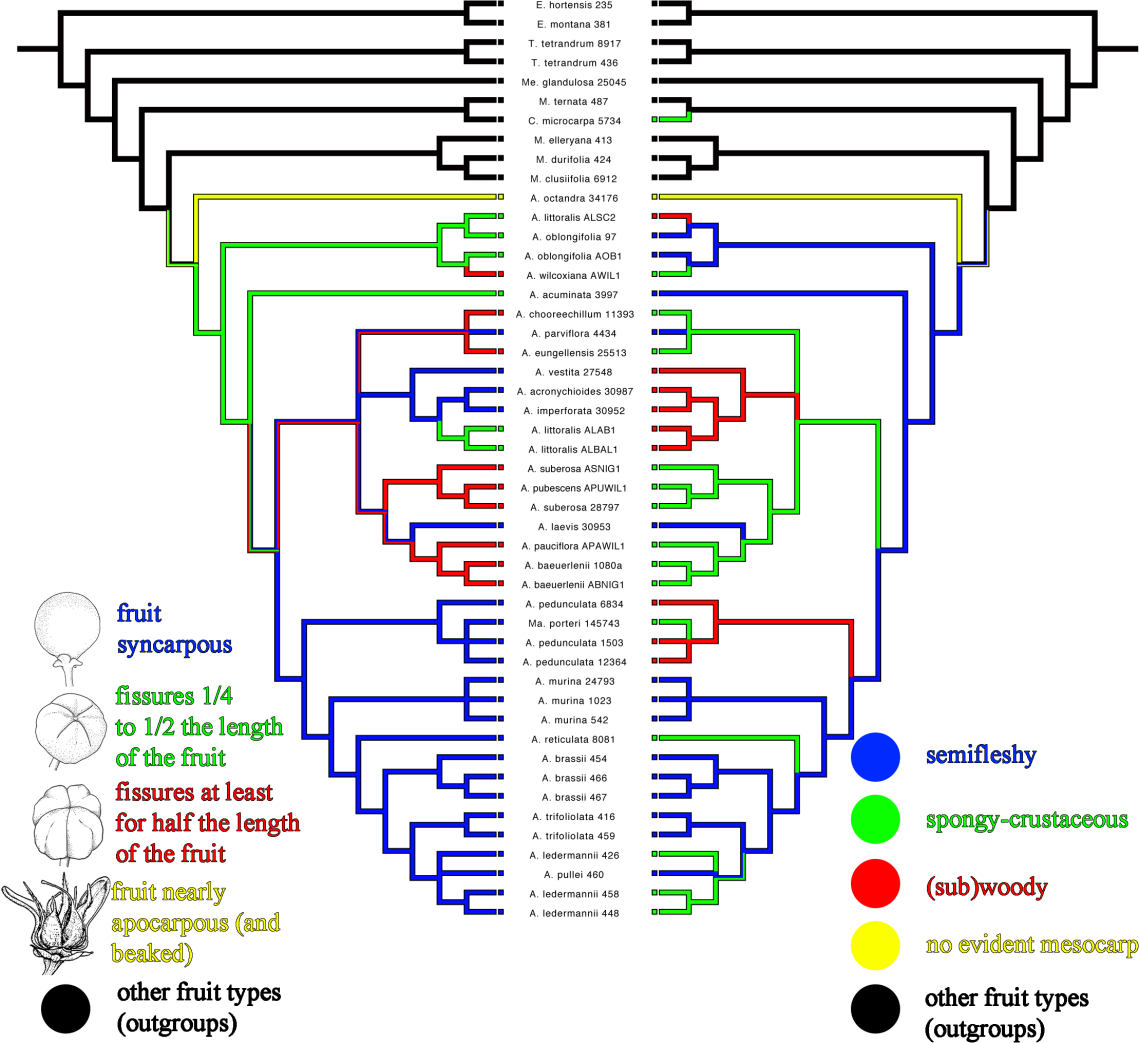
Fruit characters plotted on the 50% majority-rule consensus tree of the Bayesian analysis of the concatenated data set (Fig 1). The left tree contains the information about the fusion of carpels whereas the right tree shows the characters states for mesocarp texture. The voucher number is displayed after the species name for all taxa. Drawings by M. Appelhans (1–3) and Donald Fortesque (reprinted with permission from CSIRO). *A* = *Acronychia*; *C* = *Comptonella*; *E* = *Euodia*; *M* = *Melicope*; *Ma* = *Maclurodendron*; *Me* = *Medicosma*; *T* = *Tetractomia*.

Character mapping under parsimony indicates that fruit morphology is demonstrably homoplasious, except for the character states that are autapomorphic for *A*. *octandra* (Fig 4). Concerning the fusion of carpels, the large Australian clade (cf. Clade 4 in Fig 1) contained the whole array of character states found in the genus, excluding the autapomorphies of *A*. *octandra*. Despite the homoplasy, the degree of carpel fusion is an informative character in some subclades: the three early-branching clades within *Acronychia* (Clades 1 to 3; Fig 1) contain taxa with septicidal fissures only, whereas Clade 5 (Fig 1) comprises taxa with fully syncarpous fruits only. The character states for ‘mesocarp texture’ were scattered throughout the tree with no apparent pattern and all three states occur in the clades that contain more than one species (Clades 2, 4, 5; Fig 1).

### Biogeographical insights

**Fig 5:**
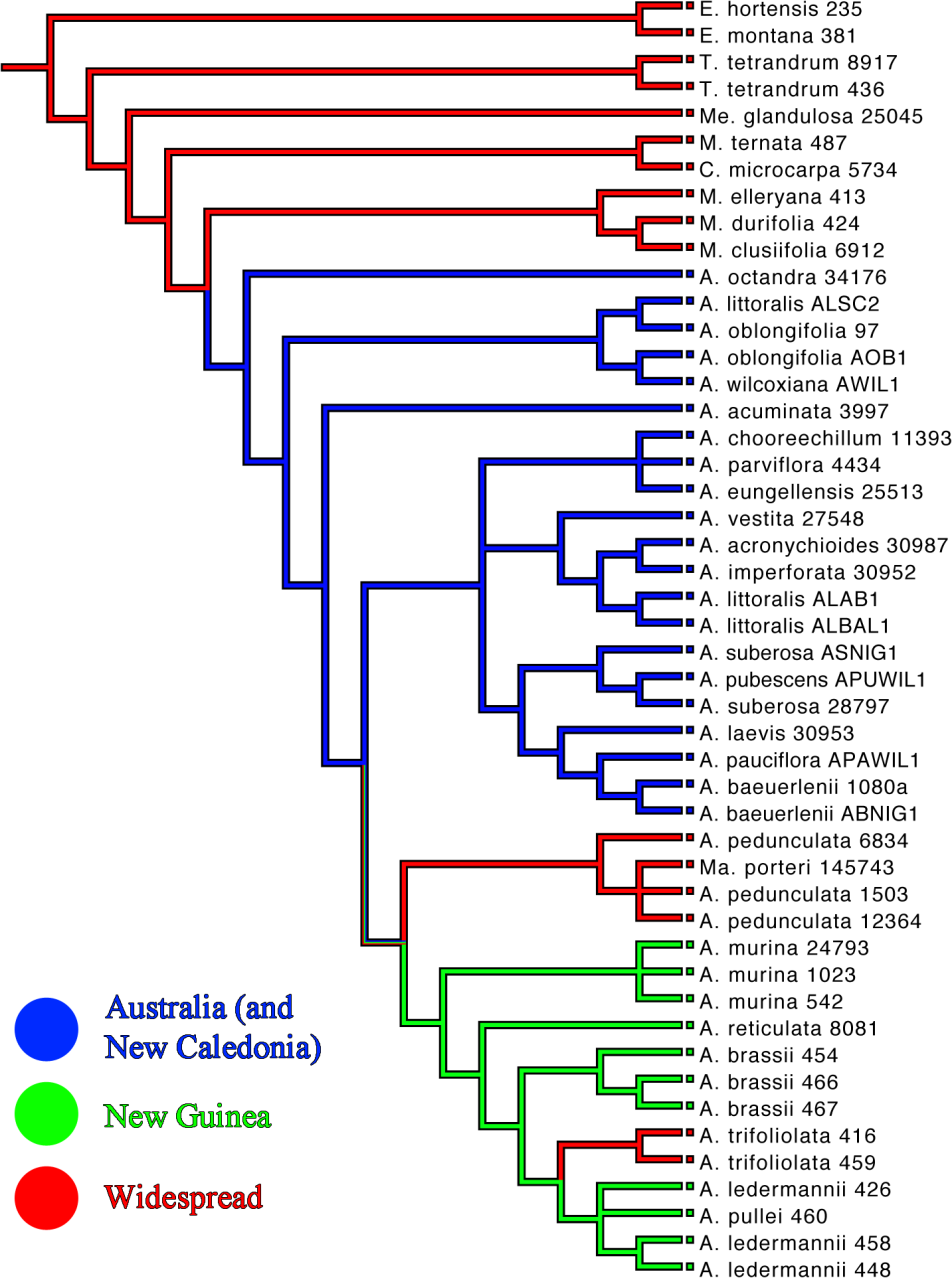
Ancestral area reconstruction in Mesquite based on the 50% majority-rule consensus tree of the Bayesian analysis of the concatenated data set (Fig 1). Voucher numbers are displayed after the species names for all taxa. *A* = *Acronychia*; *C* = *Comptonella*; *E* = *Euodia*; *M* = *Melicope*; *Ma* = *Maclurodendron*; *Me* = *Medicosma*; *T* = *Tetractomia*.

The Australian and New Guinean species are strictly separated in the phylogenetic analyses of the concatenated matrix (Fig 5) and none of the five clades contain species from both regions. All early-branching clades are endemic to Australia and this area was reconstructed as the ancestral area of *Acronychia* (Fig 5). The New Guinean clade contains two widespread subclades (*A*. *trifoliolata*; *A*. *pedunculata* + *Maclurodendron*) which are not closely related, therefore two independent colonization events to western Malesia and South-East Asia are inferred (Fig 5).

The separate ETS and ITS analyses shed further light on the biogeographical pattern of *Acronychia*. The inclusion of several additional taxa in these datasets revealed that the separation of the New Guinean and Australian species is not as strict as the analyses of the concatenated matrix suggests, since the New Guinean species *A*. *emarginata* and *A*. *rugosa* are found in an Australian clade and one accession of *A*. *acronychioides* is part of the New Guinean clade.

These first biogeographical findings should be regarded as preliminary. A more detailed analysis is only feasible in the light of a much wider taxon sampling (Appelhans et al., in preparation) since no fossil data is available for *Acronychia*. Using Mesquite for Ancestral Area Reconstruction can only deliver a broad overview, especially since the method is unable to take into account phylogenetic uncertainty, putative extinction events, incomplete taxon sampling, non-random distribution of missing taxa and varying dispersal probabilities through time (e.g. caused by tectonic movement, sea-level changes).

## Discussion

### Phylogeny and Systematics

Our study reveals that *Acronychia* is monophyletic only if *Maclurodendron* is included. This is in agreement with the study of Appelhans et al. [1], which also included one *Maclurodendron*, but far fewer *Acronychia* species than the present study. Further, the results support previous findings [1] that *Melicope* is paraphyletic with respect to *Acronychia* and other genera. Appelhans et al. [1] found that *Acronychia* was nested within *Melicope*, but support for this topology was only strong in Bayesian analyses and not in ML analyses, and only a few species of *Acronychia* were sampled. Integration of our dataset with the *Melicope* dataset of Appelhans et al. [1] will shed further light on the relationship of the two genera (Appelhans et al., in preparation).

Within *Acronychia*, *Maclurodendron* is most closely related to the widespread *A*. *pedunculata* (Fig 1). The two taxa have a largely congruent distribution. *Maclurodendron* ranges from Sumatra, Malay Peninsula, Borneo, and the Philippines to Vietnam, Hainan and Guangdong (China), and *A*. *pedunculata* ranges throughout mainland southern Asia and Malesia [1, 3, 32, 57]. *Maclurodendron* and *A*. *pedunculata* are morphologically similar in having completely syncarpous fruits and unifoliolate leaves, characters that are variable in *Acronychia*. Differences between the two taxa include unisexual vs. bisexual flowers, narrowly imbricate vs. valvate petals and differences in indumentum and shape of staminal filaments [32].

Since *Maclurodendron* is deeply nested within *Acronychia* and all *Acronychia* species have bisexual flowers, the unisexual flowers of *Maclurodendron* are interpreted as derived. Only one species of *Maclurodendron* was sampled in this study and it is therefore not clear if dioecy evolved several times from an ancestral bisexual population or if *Maclurodendron* is monophyletic within *Acronychia* and dioecy evolved only once. The differences among *Maclurodendron* species are minute and consist of differences in indumentum, sizes of floral organs, leaf texture and ornamentation of the seed coat [32]. Of these, the ornamentation of the seed coat has been regarded as taxonomically informative [32]. The seed coat of *Acronychia* has been described as smoothish to finely tuberculate, muricate or rugose ([3] p. 472), but there is no detailed description of which *Acronychia* species exhibit which ornamentation. The morphological differences among *Maclurodendron* species are minor compared to those between *Maclurodendron* and its closest relative *A*. *pedunculata*. Therefore we hypothesize a single origin of dioecy in *Maclurodendron*, to be tested with more detailed phylogenetic analysis.

The results indicate that *Maclurodendron* should be synonymized. If included in *Acronychia*, three new combinations are required (three of the six species have names available in *Acronychia*). However, a combined study of *Acronychia* and *Melicope* currently underway (Appelhans et al., in preparation) may support different generic circumscriptions in this group, therefore it would be premature to make nomenclatural changes here. Further analysis including samples of other species of *Maclurodendron* would be desirable to confirm this placement.

Three samples of *A*. *littoralis* were included in our analyses, with one sample placed in Clade 2 and the other two samples placed in Clade 4 (Fig 1). *Acronychia littoralis* consists of two different morphological and geographical types each of separate hybrid origin [50]. The parents of one type are *A*. *imperforata* and *A*. *wilcoxiana*, while the parents of the second type are *A*. *imperforata* and *A*. *oblongifolia* (which is a close relative of *A*. *wilcoxiana*) [50]. The hybrid nature of this species can be construed from the fruit morphology. *Acronychia littoralis* shares the woody mesocarp with *A*. *imperforata* and the other members of Clade 4, and subapocarpous fruits with *A*. *oblongifolia* and *A*. *wilcoxiana* (Clade 2, Fig 1). The ITS and ETS sequences proved to be by far the most variable in our study as compared to NIAi3, *trnL-trnF* and *psbA-trnH* and therefore probably have the biggest influence on tree topology. ITS and ETS were not cloned in this study and maternal haplotypes were probably sequenced for one accession and paternal haplotypes for the other, explaining why one accession of *A*. *littoralis* clusters with *A*. *wilcoxiana* and *A*. *oblongifolia*, and the other with Clade 4 (Fig 1). We performed a separate phylogenetic reconstruction excluding the *A*. *littoralis* samples in order to evaluate if the inclusion of this hybrid species has an effect on tree topology and support. Both tree topology and support values remained the same except for the relationships within Clade 2 (Fig 1; results of analysis without *A*. *littoralis* not shown).

### Fruit Evolution and Hartley´s Evolutionary Lines

Hartley [3] subdivided *Acronychia* into seven groups based on the presence or absence of septicidal fissures in the fruit, the length of the septicidal fissures and the structure of the mesocarp (fleshy and not differentiated from the exocarp; spongy-crustaceous; (sub)woody). He considered fruits with septicidal fissures and a fleshy epicarp (= exocarp + mesocarp) as the ‘primitive condition’ and fruits without septicidal fissures and spongy-crustaceous mesocarp and especially those with woody mesocarp as derived [3]. From these groupings, Hartley [3] inferred four evolutionary lines, all of which contain one or two species with the ‘primitive’ fruit characteristics. He assumed that the evolution towards the more ‘advanced’ fruit characters happened in parallel in these independent groups. Our results show that neither the fruit morphological ‘groups’ nor the ‘evolutionary lines’ are monophyletic (Figs 1 and 4). Despite this, fruit morphological characters are of phylogenetic and taxonomic significance in *Acronychia*. The three most early-branching clades of the *Acronychia* phylogeny (clades 1 to 3 in Figs 1 and 4) contain species with septicidal fissures only. *Acronychia octandra*, which is sister to the rest of the genus, is of special interest for the evolution of fruit morphology in *Acronychia*. This species has almost completely apocarpous fruits and differs from all other *Acronychia* in having a stylar beak [11]. Furthermore the exo- and mesocarp of this species is thin and chartaceous [4, 11] therefore the fruit of *A*. *octandra* may be regarded as intermediate between the capsular or follicular fruits of *Melicope* and other close relatives, and the subapocarpous or syncarpous drupes of the other *Acronychia* species. The fact that Clades 2 and 3 (Fig 1) consist of species with septicidal fissures (= subapocarpous fruits) further supports the hypothesis that syncarpous fruits in *Acronychia* are derived from an apocarpous ancestor. These results are in agreement with previous hypotheses that fleshy drupes and syncarpous gynoecia are derived character states for the *Melicope*-*Acronychia* group as well as for other Australasian Rutaceae ([2, 3, 12, 13] and literature cited in [2]). Completely syncarpous fruits have evolved several times within *Acronychia*. Clade 4 (Fig 1) contains several species with syncarpous fruits that are found in three subclades. The sampled New Guinean species and the widespread species have fully syncarpous fruits [3]. It is worth noting that while most of the unsampled species from New Guinea also have fully syncarpous fruits some do not, e.g. *A*. *goniocarpa* and *A*. *montana*.

It is striking that the *Acronychia* species with the largest distributions all have fully syncarpous fruits (without septicidal fissures) and usually a woody or subwoody mesocarp. These species include *A*. *pedunculata* and *A*. *trifoliolata* (var. *trifoliolata*), the only species occurring west of Australasia, and the Australian species *A*. *acronychioides* and *A*. *imperforata* [3, 4]. After *A*. *acronychioides* and *A*. *imperforata*, *A*. *oblongifolia* and *A*. *laevis* (which also occurs in New Caledonia), are the most widespread species in Australia. *Acronychia laevis* has fully syncarpous fruits, but its mesocarp is not differentiated from the fleshy exocarp [3, 4] and *A*. *oblongifolia* has ‘inconspicuous septicidal fissures extending for not more than half of the length’ of the fruit ([3] p. 496). In contrast, Australian species with septicidal fissures all have narrower distributions. It should be noted that not all Australian and New Guinean species with syncarpous fruits (and no septicidal fissures) are widespread: *A*. *aberrans* of north-east Queensland and *A*. *kaindiensis* of New Guinea, for example, are narrow endemics. It would appear that though having syncarpous fruits does not mean a species will be widely distributed it may predispose a taxon to being dispersed more easily. A possible explanation could be that fruits with deep septicidal fissures fall apart when they are eaten by birds, so that only some seeds are swallowed, and/or the syncarpous fruit and the (sub)woody mesocarp serves as a protective layer for the seeds. These two hypotheses need to be tested by field observations of feeding behavior and germination experiments.

Apart from *Acronychia* and *Maclurodendron*, several other genera with close affinities to *Melicope* have drupaceous fruits. These include the genera *Comptonella*, *Dutailliopsis*, *Dutaillyea*, *Picrella* and *Sarcomelicope* [1, 2, 31]. Despite the superficial similarity of the fruits, these genera have been shown, though nested within *Melicope*, not to be closely related to *Acronychia* [1, 2]. *Acronychia* differs from all of these genera in having flower buds which are longer than they are wide, from *Comptonella* and *Dutaillyea* in having a simple (vs. stellate or lepidote) indumentum, from *Comptonella*, *Dutaillyea*, *Dutailliopsis* and *Picrella* in having diplostemonous flowers (vs. haplostemonous or 4 stamens and 4 staminodes) and from *Comptonella*, *Picrella* and *Sarcomelicope* in usually having hermaphroditic (vs. functionally unisexual; note that *Maclurodendron* has unisexual flowers) flowers [3, 12, 13, 14, 31, 58, 59].

### Biogeographical insights

Mapping biogeographical areas onto the phylogenetic tree from the concatenated matrix shows that the early branching lineages within *Acronychia* exclusively contain Australian taxa and that the New Guinean and widespread species form a monophyletic group placed in a derived position within *Acronychia* (Figs 1 and 5). A geographic origin of the *Acronychia* clade in Australia is inferred and a single colonization event either via (stepping-stone) dispersal or range expansion during times of land bridges between Australia and New Guinea might have brought *Acronychia* to New Guinea. Two New Guinean species (*A*. *emarginata* and *A*. *rugosa*) were sampled in the separate ETS analyses only (Fig 2) and were placed in an Australian subclade. This suggests that the historical biogeography of *Acronychia* with respect to Australia and New Guinea is more complex. However, the lack of resolution in the ETS tree means that choosing among alternative explanatory hypotheses (e.g. vicariance, or multiple dispersals) must await further resolution of relationships.

The clear separation of Australian species from the New Guinean and widespread species is surprising given the geology of the Sahul shelf, which contains New Guinea, Australia, and nearby islands. Today, New Guinea and Australia’s Cape York Peninsula are separated by the Torres Strait, which is about 150 km wide and contains hundreds of islands, reefs and shoals [60]. Most parts of the Torres Strait are only a few meters deep and New Guinea and Australia have been connected by land bridges during glacial periods throughout the Quaternary [60, 61, 62]. Given the short distance, the many islands that could have served as stepping-stones for dispersal, the land bridges during the Quaternary, and the availability of birds as dispersal vectors, one would not expect such a clear distinction between New Guinean and Australian *Acronychia* species. On the other hand, *Acronychia* is one of many taxa for which the Torres Strait forms a relatively strict barrier, regarded by Van Steenis ([63] p. 72) as ‘one of the main demarcations of the Palaeotropic plant world’. Differences in ecology and vegetation types have been hypothesized as being the main factors of this demarcation, rather than geography [60, 61, 64, 65, 66].

New Guinean and Australian species of *Acronychia* occur in different vegetation types. Australian species occur in rain forests mostly from sea level to about 1000 m elevation though *A*. *chooreechillum* occurs in montane rain forest and shrubbery up to 1600 m elevation [4]. The majority of the New Guinean species occur in montane rain forests or cloud forests from 1500 m to 2500 m, and several species also occur in subalpine forests and shrubberies up to 3260 m [3, 5, 9]. Only four New Guinean endemic *Acronychia* species are found at elevations below 1500 m. Three of them, *A*. *dimorphocalyx*, *A*. *gurukorensis* and *A*. *reticulata* occur on the northern side of the New Guinean Highlands [3] and are thus geographically separated from the Australian species. The fourth species occurring at low elevations, *A*. *normanbiensis*, is endemic to Normanby Island in the south-east of Papua New Guinea and Hartley [3] considered this species to be closely related to *A*. *kainandiensis*, a montane species from central New Guinea. Of these four species only *A*. *reticulata* has been included in this study and it is deeply nested within the New Guinean clade. The inclusion of other species, especially *A*. *normanbiensis*, in future studies would be highly desirable.

*Acronychia* is known, at least in Australia, to be dispersed by birds including bowerbirds (green catbird, regent bowerbird, satin bowerbird), currawongs (pied currawong) and pigeons (topknot pigeon, wompoo fruit-dove, rose-crowned fruit-dove) [67, 68]. Of these, only the two fruit-doves also occur in New Guinea [69, 70]. The rose-crowned fruit-dove has been reported as a vagrant species in the Fly River delta region at sea-level. The wompoo fruit-dove is widespread in New Guinea and travels between New Guinea and Australia, but it is restricted to lowland forests from sea level to 1400 m in New Guinea [70]. The diet of the wompoo fruit-dove in New Guinea has been studied and no evidence that it eats *Acronychia* fruits has been reported [71]. Dependency on birds that do not cross the Torres Strait could be an additional factor explaining the strict phylogenetic separation of New Guinean and Australian *Acronychia* species.

Most New Guinean species of *Acronychia* have completely syncarpous fruits and only six species exhibit apical fissures like most Australian species [3, 5]. Of these six species, four are known only from the type collection [3, 5] and only one of them—*A*. *rugosa*—was included in this study. This species, together with another New Guinean endemic, *A*. *emarginata*, was found to belong to an Australian subclade (Clade 4 in Fig 1) in the ETS analyses (Fig 2) but this relationship was only supported in the ML analysis. The lack of resolution within this subclade impedes the determination of the closest relatives of these two species.

The placement of the New Guinean *A*. *emarginata* within the otherwise Australian Clade 4 is highly surprising given that it is a montane species occurring between 1760–2370 m elevation, and that it is morphologically similar to *A*. *murina* [3], a member of the New Guinean clade (Fig 1). Additional data are needed to ascertain the position of this species as well as *A*. *rugosa* and the other New Guinean species with apically fissured fruits. The position of *A*. *emarginata* in the Australian clade and the putative close relationship of the New Guinean species suggest that Torres Strait is a significant but not absolute, geographical demarcation line separating *Acronychia* lineages.

Hartley [3] hypothesized an extra-Australian origin of the Australian species *A*. *acronychioides* and *A*. *imperforata* based on an assumed close relationship with the widespread *A*. *trifoliolata* and *A*. *pedunculata* respectively. These relationships are not substantiated by our data (Fig 1) as these two Australian species are deeply nested within the Australian Clade 4 (Fig 1).

Only three *Acronychia* species, and *Maclurodendron* (six spp.), occur outside of New Guinea and Australia. *Acronychia laevis* from Australia and New Caledonia is a member of the Australian Clade 4 (Fig 1). *Acronychia pedunculata*, and to a lesser extent *Maclurodendron* range from mainland South-East Asia to New Guinea, whereas *A*. *trifoliolata* is found from Java and Christmas Island to the Solomon Islands [3, 32]. The relatively large geographical ranges of *A*. *pedunculata* and *A*. *trifoliolata* coincide with a wide variety of ecological niches occupied by these species. Both species occur from near sea level to montane elevations (up to 2200 m in *A*. *pedunculata* and 2400 m in *A*. *trifoliolata*) and a variety of vegetation types including coastal scrubs, primary and secondary rain forests, monsoon forests and montane rain forests [3]. *Maclurodendron* contains several species with narrow distributions at low elevations, and one species, *M*. *porteri*, with a larger distribution ranging from the Malay Peninsula and Sumatra to the Philippines [32]. *Maclurodendron porteri* is found over a relatively wide altitudinal range (from sea level to up to 1500 m) and in a diversity of vegetation types (primary and secondary rain forest, heath forest) indicating that the species has a wide ecological niche like *A*. *pedunculata* and *A*. *trifoliolata*.

*Acronychia pedunculata* and *Maclurodendron* form the sister clade to all other New Guinean species including *A*. *trifoliolata* (Fig 1), so that it is not clear if the *A*. *pedunculata* clade originated in Australia or if the clade split from the ancestor of the New Guinean clade after New Guinea was colonized. *Acronychia trifoliolata* is deeply nested within the New Guinean clade (Fig 1), sister to the two montane species *A*. *ledermannii* and *A*. *pullei*. *Acronychia ledermannii* was suggested to be a close relative of *A*. *trifoliolata* based on morphology [3]. Since several New Guinean species are missing in our study, the immediate affinities of *A*. *trifoliolata* cannot be finally addressed, but an origin of the species in New Guinea followed by range expansion and dispersal to the east (Solomon Islands) and west (to Java and Christmas Island) is likely. Since *A*. *pedunculata* and *A*. *trifoliolata* are not sisters, it is likely their distributions have resulted from two independent range expansions westwards from New Guinea which would add to the relatively few known cases of lineages of Australian origin dispersing across Wallace’s Line [72].

## Conclusions

This study confirms that *Acronychia* is monophyletic only if *Maclurodendron* is included. The ‘evolutionary lines’ within *Acronychia*, which are based on fruit morphological characters (connation of carpels, structure of mesocarp; [3]), are not monophyletic and should not be used to define subgenera. The early-branching clades within *Acronychia* consist of apocarpous (*A*. *octandra*) or subapocarpous species and syncarpous fruits evolved from this condition. *Maclurodendron* is most closely related to *A*. *pedunculata*. Both taxa have almost congruent distributional ranges and are morphologically similar. The dioecious flowers of *Maclurodendron* are most likely derived from a bisexual ancestor, but since only one out of six species of *Maclurodendron* was included in this study, it is not clear if dioecy in *Maclurodendron* has one or several origins. A strict separation of the Australian from the New Guinean and extra-Australasian species was inferred. Since the species north of the Torres Strait occur in different habitats compared to the Australian species, the strict separation probably has ecological rather than geographical/geological causes. The geographical origin of *Acronychia* is in Australia. Only two species of *Acronychia* occur outside of Australasia. Both species are part of an otherwise New Guinean clade, but the two species are not immediate relatives, suggesting that the colonization of areas westward of New Guinea is the result of two independent dispersal events.
